# Cell-type specific synaptic plasticity in dorsal striatum is associated with punishment-resistance compulsive-like cocaine self-administration in mice

**DOI:** 10.1038/s41386-022-01429-8

**Published:** 2022-09-07

**Authors:** Vincent Pascoli, Agnès Hiver, Yue Li, Masaya Harada, Vahid Esmaeili, Christian Lüscher

**Affiliations:** 1 Department of Basic Neurosciences, Medical Faculty, University of Geneva, 1211 Geneva, Switzerland; 2 Clinic of Neurology, Department of Clinical Neurosciences, Geneva University Hospital, 1211 Geneva, Switzerland

**Keywords:** Synaptic plasticity, Synaptic transmission

## Abstract

Addiction-related compulsion-like behavior can be modeled in rodents with drug self-administration (SA) despite harmful consequences. Recent studies suggest that the potentiation of glutamatergic transmission at the orbitofrontal cortex (OFC) to dorsal striatum (DS) synapses drives the transition from controlled to compulsion-like SA. However, the timing of the induction of this synaptic plasticity remains elusive. Here, mice were first allowed to intravenously self-administer cocaine. When mice had to endure a risk of electrical foot shock, only a fraction persevered in cocaine SA. In these persevering mice, we recorded high A/N ratios (AMPA-R/NMDA-R: α-amino-3hydroxy-5-methyl-4-isoxazolepropionic acid receptor/N-methyl-D-aspartate receptor) in both types of spiny projection neurons (i.e., D1 and D2 dopamine receptor-expressing SPNs). By contrast, when we prepared slices at the end of the acquisition period, in all mice, the A/N was high in D1R- but not D2R-SPNs. These results indicate that the transition to compulsion-like cocaine SA emerges during the punishment sessions, where synapses onto D2R-SPNs are strengthened. In renouncing individuals, the cocaine-evoked strengthening in D1R-SPNs is lost. Our study thus reveals the cell-type specific sequence of the induction of plasticity that eventually may cause compulsion-like SA.

## Introduction

With recreational cocaine consumption only a fraction of users lose control and develop a compulsive intake [1], suggesting an individual vulnerability to cocaine addiction. Indeed clinical studies report that over time one out of five users will eventually meet the diagnostic criteria of seeking and taking the drug despite major negative consequences, such as personal, social and financial loss [2–4].

The emergence of a bimodal distribution has also been observed in rodents, where compulsion-like self-administration (SA) was modeled by introducing a risk of punishment in the form of a noxious electrical foot shock. The perseverance of SA despite punishment risk was observed in about 20% of animals [5–9], a fraction similar to the one observed in humans.

Drug-evoked plasticity at specific synapses underlies behavioral adaptation such as drug-seeking and craving, preference for a drug context, and locomotor sensitization. At this early stage, addictive drugs typically evoke a potentiation of excitatory transmission onto medium-sized spiny neurons (MSNs) of the Nucleus Accumbens (NAc) [10–14] that express the dopamine D1 receptor (D1R). Upon activation of D1Rs, intracellular cascades such as the MAPK/ERK (Mitogen activate protein kinases/Extracellular signal-regulated kinases) pathway is engaged, which eventually leads to the synaptic insertion of more AMPA receptors [15, 16]. Drug exposure thus evokes synaptic plasticity selectively in D1R-MSNs *via* increased DA levels.

Compulsion-like behavior can be observed later and is associated with synaptic plasticity in the decision-making circuit. We have previously established a causal relationship between the transition to compulsion-like behavior and the strengthening of OFC to DS synapses in an addiction model based on optogenetic self-stimulation of ventral tegmental area (VTA) dopamine neurons (oDASS). Bidirectional manipulation of the synaptic strength was sufficient to enhance or attenuate compulsion-like behavior [17]. Intraperitoneal injections of cocaine may be sufficient to potentiate some OFC-DS synapses [18, 19], but only one study has examined this plasticity in compulsion-like cocaine use [20]. It is particularly intriguing that in persevering mice, synaptic potentiation is detected in both types of SPNs, given the contrasting rules of dopamine modulation of plasticity at excitatory synapses. Elevation of dopamine concentration promotes synaptic potentiation onto D1R-SPNs while promoting synaptic depression onto D2R-SPNs [21]. For this reason, the goal of the present study was to examine OFC-DST synapses with SPN-type identification before and after compulsion-like intravenous cocaine SA. Here, we provide evidence for a stepwise induction of plasticity where selective potentiation of OFC to DS synapses of D1R-SPNs emerges during the acquisition of cocaine SA in all animals, while OFC synapses onto D2R-SPNs potentiate during the punishment sessions in animals that show compulsion-like behavior.

## Materials and methods

### Animals

Mice (age 8–24 weeks) were heterozygous BAC-transgenic mice expressing tomato under D1R control (Drd1a-td-tomato). To identify D2R-SPNs, Adora2a-cre was used (Jackson Laboratories). Weight and sex were distributed homogeneously among the groups. All transgenic mice were backcrossed into C57BL/6 for four or more generations. All animals were kept in a temperature- and humidity-controled environment with an inverted 12h-light/12h-dark cycle (lights on at 7:00 pm). All procedures were approved by the Institutional Animal Care and Use Committee of the University of Geneva and by the animal welfare committee of the Cantonal of Geneva, in accordance with Swiss law.

### Surgery

#### Jugular vein catheter

Implantations were made as previously described [11]. Successful placement was confirmed by withdrawing blood from the vein. Mice were allowed to recover for 3–5 days before the start of drug SA training and received paracetamol (2 mg/ml po) and Amikin (1 mg/kg sc). Catheters were flushed daily with heparin before and after each session. For 8 out of 68 mice, the catheter patency had degraded before the end of the experiment and data were therefore not obtained for these mice.

#### Stereotaxic infusions

Injections were carried out as previously described [17]. Adeno-associated virus AAV8-hSyn-Chrimson-GFP (Duke Vector Core), was injected bilaterally in the OFC (AP +2.6; ML ±1.75; DV −1.95). Anesthesia was induced with 5% isoflurane and maintained at 2.5% (w/v) (Baxter AG). AAV5-Ef1α-DIO-mCherry (UNC GTC Vector Core), was injected bilaterally in the DS (AP +0.8; ML ±1.65; DV −3.3) of Tg(Adora2a-cre)2MDkde mice. Virus injections (0.5 μl) were made with graduated pipettes (Drummond Scientific Company), broken back to a tip diameter of 10–15 μm, at an infusion rate of 0.05 μl/min. Three screws drilled into the skull fixed the implant and secured with dental cement.

### Behavioral testing

#### Acquisition of cocaine self-administration (SA)

SA was performed during the dark phase of the inverted light/dark cycle, in mouse operant chambers (ENV-307A-CT; Med Associates). Two retractable levers were present on both sides of one wall of the chamber. A cue-light was located above each lever. During intravenous saline or cocaine SA sessions, the stainless-steel tubing of the catheter device was connected through a CoEx PE/PVC tubing (BCOEX-22, Phymep) to a 22GA swivel (375/22PS, Instech Solomon) and then an infusion pump (PHM100; Med-Associates). The apparatus was controlled, and data captured with MED-PC IV (Med-Associates). Each 300 min session started with the insertion of the two levers into the operant chamber. During 4 sessions, a single press (Fixed ratio 1, FR1) on the active lever resulted in an infusion of saline (0.9% saline delivered at 0.0177 ml/s as a unit volume depending on the weight of the mouse) or 0.75 mg/kg of cocaine (cocaine hydrochloride, Geneva University Hospital, dissolved in 0.9% saline at 0.75 mg/ml and delivered at 0.0177 ml/s as a unit dose depending on the weight of the mouse) paired with 10 flashes of 1 s duration of the cue light above the active lever. From day 5–8 the fixed ratio was increased to 2 (FR2) and from day 9 to 12 fixed ratio was increased to 3 (FR3). Rewarded lever press, at completion of the FR, triggers a timeout period of 20 s during which cocaine was no longer available. The active lever (left or right lever) was randomly assigned for each mouse.

#### Cocaine or saline SA under progressive ratio schedule

Between acquisition session 11 and 12, mice were subjected to a progressive ratio session. The breakpoint was taken as the last schedule reached, either after 4 h or after 40 min had elapsed without lever pressing. The reinforced schedules were as follows: 1, 3, 5, 8, 12, 16, 22, 29, 38, 50, 65, 84, 108, 139, 178, 228, 291, 371, 473, 603, 767, 977, 1243 and 1582. The total number of active lever presses was taken as a measure of perseverance (Supplemental Fig. 1).

#### Cocaine SA under punishment risk

Three unpunished sessions of 2 hours served as a baseline. In subsequent session every third completion of the FR3 a foot shock (500 ms, 0.2 mA) was applied immediately after the rewarded active lever press. In addition, a second cue (house light) predicting the oncoming shock was paired with the second lever press of the FR3 schedule. Perseverance rate was calculated as the ratio of mean number of infusions from the last two punishment sessions by mean number of infusions during the three baseline sessions.

### Slice electrophysiology

Whole-cell patch-clamp recordings of striatal neurons were performed 24 h after the last punished session, after acquisition or in slices from naive mice. Ex vivo synaptic responses to optogenetic stimulation of OFC terminals of the striatal neurons were measured with visualized whole-cell patch-clamp recording technique. Coronal 230 μm slices of mouse brain were prepared in cooled artificial cerebrospinal fluid containing (in mM): NaCl 119, KCl 2.5, MgCl_2_ 1.3, CaCl_2_ 2.5, Na_2_HPO_4_ 1.0, NaHCO_3_ 26.2 and glucose 11, or CsCl 130, NaCl 4, creatine phosphate 5, MgCl_2_ 2, NA_2_ATP 2, NA_3_GTP 0.6, EGTA 1.1, HEPES 5, spermine 0.1 and QX-314 (5 mM) bubbled with 95% O_2_ and 5% CO_2_. Slices were kept at 32–34 °C in a recording chamber superfused with 2.5 ml/min artificial cerebrospinal fluid. Direct pathway (D1R-SPNs) and indirect pathway (A2aR/D2RSPNs) of the striatum were identified by the presence or absence of the td-tomato in BAC transgenic mice or mCherry expression in Tg(Adora2a-cre)2MDkde mice by using a fluorescence microscope (Olympus BX50WI, fluorescent light U-RFL-T) and confirmed on confocal images of the recorded neuron filled with biocytin (Sigma, B4261) and stained with streptavidin–Cy5 (Invitrogen, 434316). Currents were evoked with optogenetic stimulation of OFC terminals infected with Chrimson. In BAC transgenic (Drd1a–td-tomato) or Tg(Adora2acre)2MDkde, the OFC was infected with AAV8-hSyn-Chrimson-eYFP and tdtomato+, mCherry+, td-tomato- or mCherry-cells were filled with biocytin and identification was confirmed post-hoc on confocal images. The AMPAR/NMDAR ratio (A/N ratio) was calculated with amplitude of EPSCs recorded at +40 mV. AMPAR-EPSC component was pharmacologically isolated using NMDA antagonist D-2-amino-5-phosphonovaleric acid (D-AP5, 50 μM) and the NMDAR-EPSC component was determined by subtraction. The holding potential was -70 mV and the access resistance was monitored by a hyperpolarizing step of −4 mV. The liquid junction potential was small (−3 mV), and traces not corrected. Experiments were discarded if the access resistance varied by more than 20%. Currents were amplified (Multiclamp 700B, Axon Instruments), filtered at 5 kHz and digitized at 20 kHz (National Instruments Board PCI-MIO-16E4, Igor, Wave Metrics). The rectification index of AMPAR was calculated as the ratio of the chord conductance calculated at negative potential divided by chord conductance at positive potential. The paired-pulse ratio (PPR) was measured at the beginning of the recordings by delivering 2 pulses of 4 ms with a 76 ms interval. Examples traces are averages of 10–15 sweeps. Spontaneous EPSCs (sEPSCs) recorded at negative potential (-70 mV) were extracted from the 50 first sweeps of the previous recorded neurons. Analysis was performed on a minimum of 300 events. All experiments were performed in the presence of picrotoxin (100 μM).

### Imaging

Slices used for electrophysiology were post-fixed overnight in a solution containing 4% (w/v) paraformaldehyde in PBS (pH 7.5) and stored at 4 °C. Amplification of the Drd1a-td-tomato was performed with immunohistochemistry, the following primary antibody (rabbit polyclonal Anti-RFP, MDL PMPP5; lot047, diluted 1:1000) and secondary antibody (donkey anti-rabbit Cy3, Millipore AP182C, lot 3382285, diluted 1:500). For biocytin (Sigma-Aldrich, B4261) staining, streptavidin–Cy5 (Invitrogen 434316) was used. Nuclei were stained with Hoechst (Sigma-Aldrich) and slices were mounted with Mowiol (Sigma-Aldrich). High-magnification confocal images were obtained using sequential laser scanning confocal microscopy (Zeiss LSM800). Photomicrographs were obtained with the following band-pass and long-pass filter settings: UV excitation (band-pass filter: 365/12 nm), GFP (band-pass filter: 450–490 nm), Cy3 (band-pass filter: 546/12 nm) and Cy5 (band-pass filter: 546/12 nm).

### Statistical analysis

Multiple comparisons were first subject to mixed-factor ANOVA defining both between- and/or within-group factors. Where significant main effects or interaction terms were found (*P* < 0.05), further comparisons were made by a two-tailed Student’s *t*-test with Bonferroni corrections applied when appropriate (that is, the level of significance equaled 0.05 divided by the number of comparisons). For comparison of self-administration across sessions a mixed ANOVA for repeated measures was used and where significant main effects or interaction terms were found (*P* < 0.05), further comparisons were made by a post-hoc comparison with Dunnett’s. Pearson’s *r* was used to test significant correlations. One-way ANOVA was used for multiple group comparison and where significant effect was found (*P* < 0.05), further comparisons were made by a post-hoc comparison Tukey. Single comparisons of between- or within-group measures were made by two-tailed nonpaired or paired Student’s *t*-test, respectively.

### Behavioral clustering analysis

We used a clustering algorithm on the entire set of behavioral variables to identify renouncing and persevering mice. Clustering analysis was performed using custom-written codes in Matlab (Mathworks). The behavioral variables consist of the rate of all active lever presses, all inactive lever presses, active lever presses during timeout periods, inactive lever presses during timeout periods, and infusions. Timeout periods lasted for 20 s following each cocaine infusion. Only variables during the last two days of punishment (P3 and P4) were included in the clustering analysis. Prior to clustering, the behavioral variables of each mouse were normalized to their mean value during the baseline sessions (B1 to B3). Then, in an iterative manner, a nonlinear dimension reduction algorithm (t-distributed stochastic neighbour embedding, first 3 dimensions) together with hierarchical clustering (Matlab functions ‘pdist’, ‘linkage’ and ‘cluster’ with a metric = seuclidean and linkage = ward) was performed 100 times, and the best tree was taken based on the clustering robustness, namely the mean silhouette score and the Cophenetic distance. Behavioral variables during the baseline sessions (B1 to B3) and the first two punishment days (P1 and P2), together with perseverance, were plotted for visualization but were not considered during clustering.

## Results

### Plasticity at OFC to DS synapses with compulsion-like cocaine SA

**Fig. 1:**
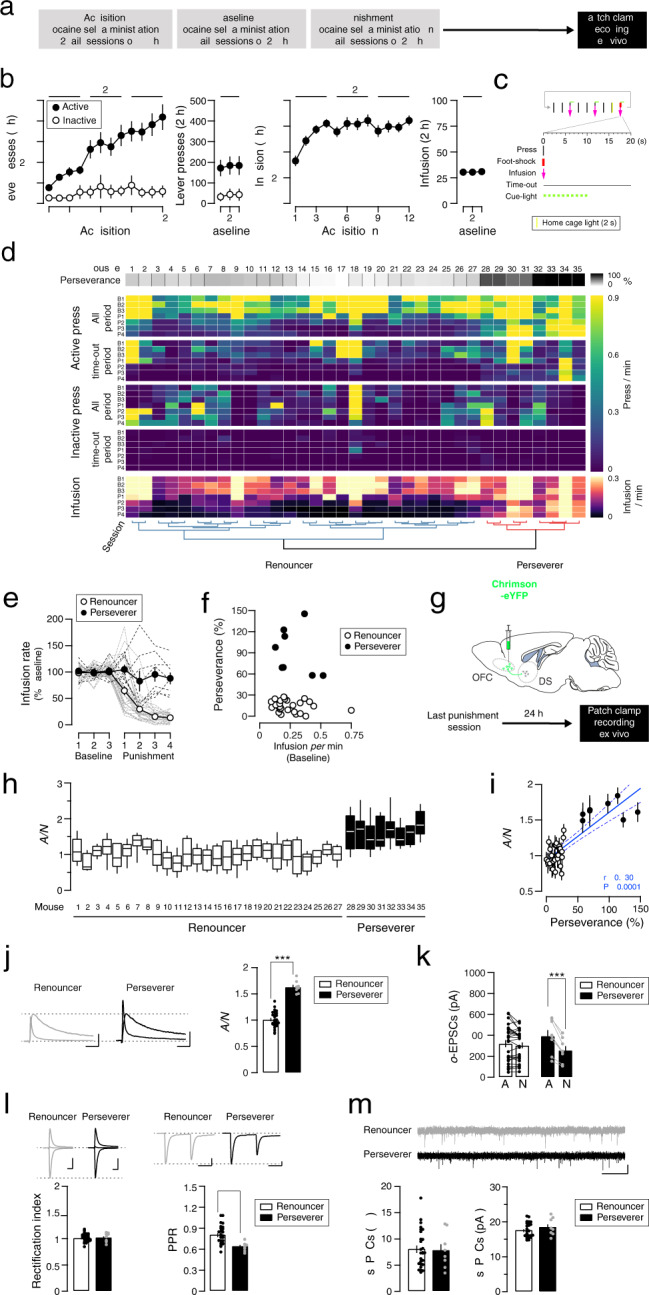
Potentiation of OFC-DS synapses correlates with perseverance when cocaine self-administration is associated with a risk of foot shock. **a** Experiment schedule. **b** Active and inactive lever presses (left) and infusion (right) during acquisition and baseline sessions of cocaine self-administration (*N* = 35 mice). **c** Schedule of punished sessions with foot shock (0.2 mA, 500 ms) every third FR3 completion. **d** Heat maps showing the behavioral parameter of each mouse during baseline and punished sessions. Clustering was performed with all behavioral parameters of the last two punished sessions and correlated with perseverance. **e** Rate of cocaine infusions during punished sessions normalized to baseline sessions in renouncers and perseverers (*N* = 27 and 8 mice respectively). Dashed lines represented individual mouse. **f** Perseverance as a function of baseline cocaine infusion rate. Each dots represents a mouse. **g** Slice recordings 24 h after the last punished session in mice infected with Chrimson-eYFP in the OFC. **h** Box plot of the distribution (median, 25%, 75%, minimal and maximal values) of the ratios of optogentically evoked AMPAR to NMDAR-EPSCs (A/N) recorded at positive potential (+40 mV) from several cells (total of 272 recorded cells/35 mice, with 4–13 cells/mouse) in each renouncing or persevering mice (*N* = 27 and 8 mice). **i** Mean AMPAR/NMDAR (A/N) ratio as a function of perseverance, per animal and correlation (Pearson’s *r* = 0.843, *P* < 0.001; *N* = 35 mice). **j** Example traces (average of 15 sweeps) of AMPAR- and NMDAR-EPSCs recorded at +40 mV. Scale bars, 50 ms, 50 pA. Grouped data for A/N ratio (*N* = 27 and 8 mice, Mean of 5–13 cells/mouse). Student’s *t*-test: ***P < 0.001. **k** Mean amplitude of AMPAR- and NMDAR-EPSCs evoked with optogenetic stimulation (o-EPSCs) of OFC terminals in slices per mouse (*N* = 27 and 8 mice). Bonferroni post-test ***P < 0.001, for AMPAR vs NMDAR amplitude in perseverers. **l** Example traces (average of 15 sweeps) of AMPAR-EPSCs recorded at −70, 0 and +40 mV and rectification index in renouncers and perseverers (*N* = 27 and 8 mice, total of 272 recorded cells/35 mice, Mean of 4–13 cells/mouse). Scale bars, 20 ms, 250 pA. Example traces (average of 3 sweeps) of two successive AMPAR-EPSCs with an inter-pulse interval of 76 ms recorded at -70 mV and paired pulse ratio (PPR) in renouncers and perseverers (*N* = 27 and 8 mice, total of 292 recorded cells/ 35 mice, Mean of 4–13 cells/mouse). Student’s *t*-test: *P < 0.05. Scale bars, 50 ms, 200 pA. **m** Example traces (1 sweep) of spontaneous EPSCs (sEPSCs) recorded at -70 mV. Frequency and amplitude of sEPSCs in renouncers and perseverers (*N* = 27 and 8 mice, total of 265 recorded cells/35 mice, Mean of 4–13 cells/mouse). Scale bars, 500 ms, 20 pA. Data are expressed as mean ± SEM. FR fixed-ratio, AMPAR α-amino-3-hydroxy-5-methyl-4-isoxazolepropionic acid receptor, NMDAR N-methyl-D-aspartate receptor, EPSC excitatory postsynaptic current, SA self-administration, OFC Orbitofrontal cortex, DS Dorsal Striatum, PPR paired-pulse ratio.

First, mice learned to self-administer cocaine (iv, 0.75 mg/kg/infusion) in 12 daily sessions of 5 h while increasing the fixed ratio (FR) every 4 days. Next, the mice had 3 sessions of 2 h, which we took as a baseline, and then underwent the 4 sessions where every third injection was punished by an electric shock (0.2 mA, 500 ms). This behavioral assessment was followed by ex vivo slice recordings (Fig. 1a). During the acquisition of cocaine SA, the number of active lever presses increased to match the increase in FR, to maintain a steady cocaine intake (Fig. 1b). This shows that the mice learned the contingency between pressing the lever and receiving cocaine. The mice also discriminated the active from the inactive lever. The rate of presses was maintained during the subsequent 3 baseline sessions of 2 h. The number of infusions remained stable after 3 days of acquisition until the end of the 3 baseline sessions (Fig. 1b). For the punishment sessions, every third infusion was preceded by the illumination of the house light to predict a foot-shock contingent to the third press of the FR completion. Every FR completion led to the illumination of a cue light, triggered the intravenous infusion, and a timeout period of 20 s (Fig. 1c). The behavioral analysis yielded two groups of 8 punishment-resistant and 27 punishment-sensitive mice (Fig. 1d). In line with our previous publications, we will call these groups perseverers and renouncers, respectively [17, 20].

The infusion rate in the renouncing individuals was strongly reduced in sessions with punishment risk compared to baseline sessions, while persevering mice continued unabated (Fig. 1e). There was no correlation between the rate of cocaine SA at baseline and the perseverance of cocaine SA under punishment risk (Fig. 1f). We retrospectively analyzed the acquisition of cocaine self-administration behavior and found no difference between renouncing and persevering mice (Supplemental Fig. 1a–c). The cocaine infusion rate was similar between renouncers and perseverers (mixed ANOVA for repeated measures: Sessions effect, *F*_(3, 99)_ = 2.12, *P* = 0.127; group effect, *F*_(1, 33)_ = 0.81, *P* = 0.374; Sessions x group effect, *F*_(3, 99)_ = 0.03, *P* = 0.995) (Supplemental Fig. 1d). The breakpoint determined in a progressive ratio session was also similar between renouncers and perseverers (Student’s *t*-test: *t*_(19)_ = 0.071, *P* = 0.944) and did not correlate with the perseverance (Supplemental Fig. 1f). Since we infected the animals prior to SA with the excitatory opsin Chrimson-R2 in the OFC, we prepared slices of the DS the day after the last punishment session (Fig. 1g). We first analyzed the synaptic transmission recordings blind to the cell-type. We assessed the strength of OFC to DS synapses by recording EPSCs mediated by AMPAR and NMDAR, which allowed us to calculate the ratio of AMPAR to NMDAR components (A/N). The goal was to record from SPNs of every mouse and correlate the mean A/N with its behavior (206 SPNs from 35 animals, 4–13 SPNs/mouse). The distribution of A/N ratio collected from each mouse is represented to appreciate the cell-by-cell variability and the median is calculated for every mouse. The distribution and the median value of A/N of renouncing and persevering mice seemed different even before grouped data analysis (Fig. 1h). In addition, we found a correlation (Pearson’s *r* = 0.830, *P* < 0.001; *N* = 35 mice) between the mean A/N of each mouse and its perseverance score (Fig. 1i). Indeed a higher A/N ratio was found in perseverers (Student’s *t*-test: *t*_(33)_ = 10.81, *P* < 0.0001), indicating that OFC to DS synapses were strengthened only in mice exhibiting compulsion-like behavior (Fig. 1j). Even though the absolute amplitude of AMPAR- and NMDAR-EPSCs depends on the number of synapses stimulated–a parameter difficult to control–we found a trend for higher amplitudes of optogenetically evoked AMPAR-EPSCs in perseverers while NMDAR-EPSCs were identical. As a result, the AMPAR-EPSCs amplitude was larger than NMDAR-EPSCs amplitude in perseverers but not in renouncers (mixed two-way ANOVA: EPSCs amplitude effect, *F*_(1, 33)_ = 48.43, *P* < 0.0001; group effect, *F*_(1, 33)_ = 0.05, *P* = 0.82; EPSCs amplitude x group effect, *F*_(1, 33)_ = 29.87, *P* = 0.0001; Bonferroni post-test: *t*_(7)_ = 7.07, *P* < 0.0001 for AMPA *vs* NMDA in perseverers). This suggests that the higher A/N ratio in perseverers indeed reflects a synaptic potentiation recruiting more AMPARs, leaving NMDARs unaffected (Fig. 1k). There was no evidence for a change of the AMPAR subunit composition as the current/voltage relationship was linear in both, renouncers and perseverers, as shown by the rectification index (Student’s *t*-test: *t*_(33)_ = 0.37, *P* = 0.712). The paired-pulse ratio (PPR) was slightly reduced in persevering mice (Student’s *t*-test: *t*_(31)_ = 3.26, *P* = 0.0027), suggesting a higher release probability at OFC terminals to DS (Fig. 1l). Neither amplitude nor frequency of spontaneous EPSCs (sEPSCs) recorded from the same cells were affected (Student’s *t*-test: *t*_(33)_ = 1.05, *P* = 0.301 and *t*_(33)_ = 0.19, *P* = 0.850, for frequency and amplitude, respectively) (Fig. 1m). Overall, our data confirm a synaptic strengthening of OFC to DS synapses linked to the perseverance of cocaine self-administration despite punishment risk, similar to the observations made in the oDASS model [17, 20].

### Strengthening of synapses from OFC onto D1R- and D2R-SPNs with compulsion-like cocaine SA

**Fig. 2:**
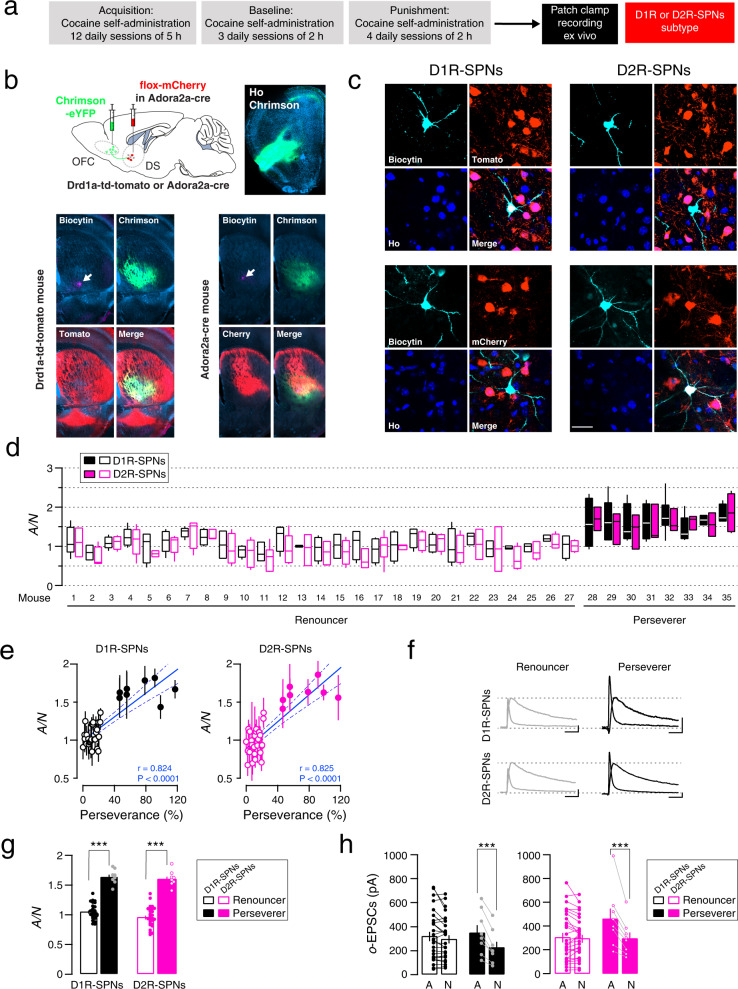
Cell-type specific synaptic potentiation in compulsion-like mice. **a** Experiment schedule. **b** Post-hoc identification of direct and indirect pathways SPNs. Slice recordings 24 h after the last punished session in mice infected with Chrimson-eYFP in the OFC. SPNs of the DS from BAC Drd1a- td-tomato transgenic mice and Adora2a-cre mice injected with AAV5-Ef1a-DIO-mCherry were filled with biocytin (revelation with streptavidin-Cy5, cyan). **c** Examples of neurons recorded and filled with biocytin (revelation with streptavidin-Cy5, cyan), considered from the direct pathway (D1R-SPNs) either positive in Drd1a-td-tomato mouse (upper left) or negative in Adora2a-cre/flox-cherry mouse (bottom left). Negative neuron from Drd1a-td-tomato mouse (upper right) or positive neuron in Adora2a-cre/flox-cherry mouse (bottom right), conversely considered from the indirect pathway (D2R-SPNs). Scale bar, 50 µm. **d** Box plot of the distribution (median, 25%, 75%, minimal and maximal values) of the ratios of optogentically evoked AMPAR to NMDAR-EPSCs (A/N) recorded at positive potential (+40 mV) from D1R- and D2R-SPNs (total of 135 D1R-SPNs and 137 D2R-SPNs/35 mice, with 2–8 identified cells/mouse) in each renouncing or persevering mice (*N* = 27 and 8 mice respectively). **e** Mean AMPAR/NMDAR (A/N) ratio as a function of perseverance, per animal and correlation (For D1R-SPNs Pearson’s *r* = 0.855, *P* < 0.001; *N* = 35 mice; For D2RSPNs Pearson’s *r* = 0.815, *P* < 0.001; *N* = 35 mice). **f** Example traces (average of 15 sweeps) of AMPAR- and NMDAR-EPSCs recorded at +40 mV in D1R-or D2R-SPNs from renouncer and perseverer. Scale bars, 50 ms, 50 pA. **g** A/N ratio from D1R-or D2R-SPNs of renouncers and perseverers (*N* = 27 and 8 mice, mean of 2–8 identified cells / mouse). Bonferroni post-test ***P < 0.001 for Renouncers *vs* Perseverers in D1R-SPNs or D2R-SPNs. **h** Mean amplitude of AMPAR- and NMDAR-EPSCs evoked with optogenetic stimulation of OFC terminals (o-EPSCs) to D1R-or D2R-SPNs in slices *per* mouse (*N* = 27 and 8 mice). Bonferroni post-test **P < 0.01 or ***P < 0.001, for AMPAR vs NMDAR amplitude of perseverers in D1R-and D2R-SPNs, respectively. Data are expressed as mean ± SEM. SPNs Spiny Projection Neurons, Ho Hoechst, OFC Orbitofrontal cortex, DS Dorsal Striatum.

Since dopamine plays a role in the modulation of excitatory synapses transmission, and D1 and D2 dopamine receptors couple to distinct sets of G proteins, cocaine may drive cell-type specific forms of synaptic plasticity in SPNs, as it has been observed in MSNs of the NAc. We, therefore, re-analyzed the electrophysiological data in the light of the cell-type identity (Fig. 2a). To visualize D1 and D2R-SPNs during recording and *post-hoc* confocal images, we used Drd1a-td-tomato, or Adora2a-cre mice injected with Chrimson in the OFC. We also injected Adora2a-cre mice with an adenovirus providing mCherry in the DS. We then filled the cells with biocytin through the patch pipette for *post-hoc* cell-type confirmation (Fig. 2b). The presence of biocytin in a D1rd1a-td-tomato positive recorded cell or mCherry negative cell in Adora2a-cre mice was taken as D1R-SPN. Conversely, the presence of biocytin in a D1rd1a-tdtomato negative recorded cell or mCherry positive cell in Adora2a-cre mice was taken as D2R-SPNs (Fig. 2c). The few interneurons patched were discarded based on their intrinsic electrophysiological properties [22]. We first separate D1R- and D2R-SPNs for each mouse to appreciate the distribution, the cell-by-cell variability, and the median of A/N ratio collected from each mouse. Overall and before grouped data analysis, the distribution and the median value of A/N seemed similar in D1R and D2R-SPNs for every mouse, although renouncing and persevering mice appeared to be different for both D1R and D2R-SPNs (Fig. 2d). In addition, we found a correlation (Pearson’s *r* = 0.824, *P* < 0.001 and *r* = 0.825, *P* < 0.001, for D1R and D2R-SPNs, respectively; *N* = 35 mice) between the mean A/N recorded from both of D1R and D2R-SPNs in each mouse and its perseverance score (Fig. 2e). Indeed a higher A/N ratio was found for perseverers in both D1R- and D2R-SPNs (mixed two-way ANOVA: SPNs-type effect, *F*_(1,66)_ = 2.13, *P* = 0.149; group effect, *F*_(1,66)_ = 206.6, *P* < 0.0001; SPNs-type x group effect, *F*_(1,66)_ = 0.535, *P* = 0.467; Bonferroni post-test: *t*_(33)_ = 9.65, *P* < 0.0001 for Per. *vs* Ren. in D1R-SPNs; *t*_(33)_ = 10.68, *P* < 0.0001 for Per. *vs* Ren. in D2R-SPNs) indicating that OFC to DS synapses were strengthened in persevering mice for both D1R- and D2R-SPNs (Fig. 2f-g). We also analyzed the absolute amplitude of AMPAR- and NMDAR-EPSCs evoked with optogenetic stimulation and found again higher AMPAR-EPSCs in perseverers while NMDAR-EPSCs were identical. In fact, AMPAR-EPSCs amplitude were higher than NMDAR-EPSCs amplitude in perseverers but not in renouncers in D1R-SPNs (mixed two-way ANOVA: EPSCs effect, *F*_(1,33)_ = 40.01, *P* < 0.0001; group effect, *F*_(1,33)_ = 0.074, *P* = 0.787; EPSCs-type x group effect, *F*_(1,33)_ = 18.18, *P* = 0.0002; Bonferroni post-test: *t*_(7)_ = 6.03, *P* < 0.0001 for AMPA-EPSCs vs NMDA-EPSCs in perseverers) and D2R-SPNs (mixed two-way ANOVA: EPSCs effect, *F*_(1,33)_ = 39.46, *P* < 0.0001; group effect, *F*_(1,33)_ = 1.02, *P* = 0.32; EPSCs-type x group effect, *F*_(1,33)_ = 31.41, *P* < 0.0001; Bonferroni post-test: *t*_(7)_ = 6.766, *P* < 0.0001 for AMPA-EPSCs vs NMDA-EPSCs in perseverers). These results suggest that the higher A/N ratio in perseverers indeed reflects a synaptic potentiation in both cell-type of the DS (Fig. 2h). Taken together, synapses at OFC to both types of SPNs in the DS were strengthened in mice that self-administered cocaine despite the risk of foot shock when compared to renouncing mice one day after the last punishment session.

### Synaptic transmission after the acquisition of cocaine SA with no punishment risk

**Fig. 3:**
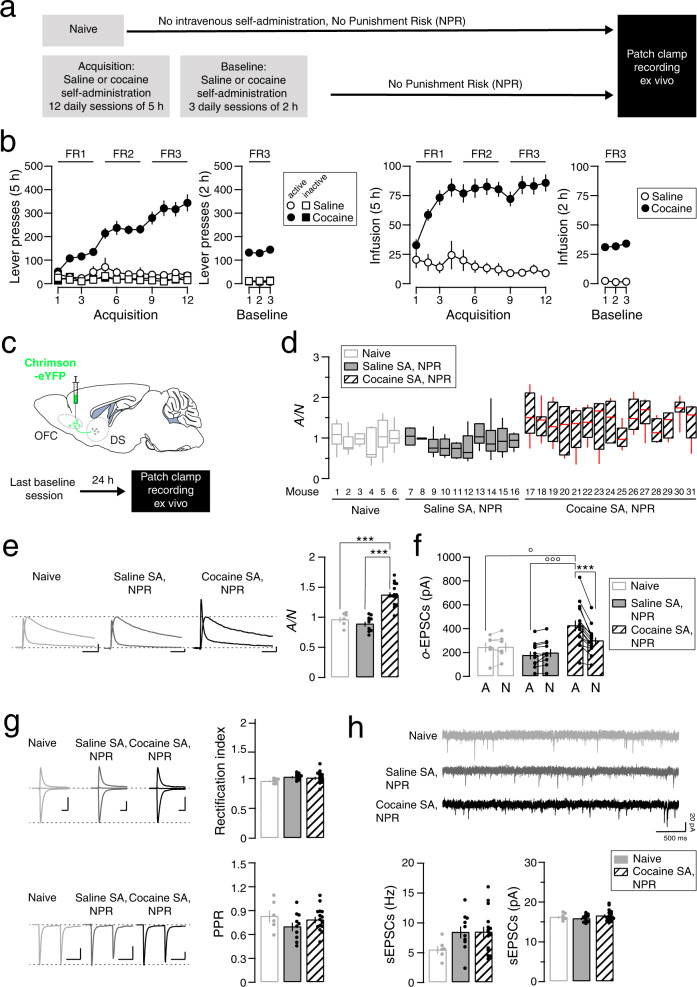
Synaptic transmission at OFC-DS synapses after acquisition of cocaine SA. **a** Experiment schedule. **b** Active and inactive lever presses (left) and infusion (right) during acquisition and baseline sessions of saline or cocaine self-administration (*N* = 10 and 15 mice, respectively). **c** Slice recordings 24 h after the last baseline session in mice infected with Chrimson-eYFP in the OFC. **d** Box plot of the distribution (median, 25 %, 75 %, minimal and maximal values) of the ratios of optogentically evoked AMPAR to NMDAR-EPSCs (A/N) recorded at positive potential (+40 mV) from several cells (total of 215 recorded cells/31 mice, with 2–10 cells/mouse) in mice that were naive or underwent saline or cocaine SA but did not experience punishment (Saline or Cocaine SA, NPR, *N* = 6, 10 and 15 mice respectively). **e** Left, example traces (average of 15 sweeps) of AMPAR- and NMDAR-EPSCs recorded at +40 mV. Scale bars, 50 ms, 50 pA. Right panel, grouped data for A/N ratio (*N* = 6, 10 and 15 mice, mean of 2–10 cells/mouse). Tukey post-test: ***P < 0.001. **f** Mean amplitude of AMPAR- and NMDAR-EPSCs evoked with optogenetic stimulation (o-EPSCs) of OFC terminals in slices *per* mouse (*N* = 6, 10 and 15 mice). Bonferroni post-test ***P < 0.0001, for AMPAR vs NMDAR amplitude in mice that underwent cocaine SA without punishment session; °*P* < 0.001, for oAMPAR-EPSCs amplitude from naive vs cocaine SA, NPR; °°°*P* < 0.001, for oAMPAR-EPSCs amplitude from saline vs cocaine SA, NPR. **g** Example traces (average of 15 sweeps) of AMPAR-EPSCs recorded at −70, 0, and +40 mV and rectification index in naive mice and mice that underwent cocaine SA (*N* = 6, 10, and 15 mice, 215 cells from 31 mice, mean of 2–10 cells/mouse). Scale bars, 20 ms, 250 pA. Example traces (average of 3 sweeps) of two successive AMPAR-EPSCs with an inter-pulse interval of 76 ms recorded at −70 mV and paired-pulse ratio (PPR) in naive and saline or cocaine self-administering mice, no punishment risk associated (*N* = 6, 10 and 15 mice, 268 cells from 31 mice, mean of 3–18 cells/mouse). Scale bars, 50 ms, 200 pA. **h** Example traces (1 sweep) of spontaneous EPSCs (sEPSCs) were recorded at −70 mV. Frequency and amplitude of sEPSCs in naive and saline or cocaine SA, NPR (*N* = 6, 10, and 15 mice, 236 cells from 31 mice, mean of 4–12 cells/mouse). Scale bars, 500 ms, 20 pA. Data are expressed as mean ± SEM. NPR, No punishment risk.

To parse the moment of the synaptic strengthening, we next prepared a new cohort of mice to record electrophysiological parameters the day following the last baseline session of cocaine or saline SA, with no punishment risk (NPR) (Fig. 3a). For electrophysiological parameters, these groups were also compared to naive mice. During acquisition and baseline sessions of cocaine SA, the rate of infusions and lever presses in this distinct cohort (*N* = 15) were similar to the initial cohort (*N* = 35), while mice with offered saline (*N* = 10) rarely pressed the lever during acquisition and baseline sessions (Fig. 3b, Supplemental Fig. 2b). The breakpoint was significantly higher for cocaine than for saline SA (Student’s *t*-test: *t*_(16)_ = 3.22, *P* = 0.0053) (Supplemental Fig. 2c). We then optogenetically targeted the OFC to DS projection (Fig. 3c) and recorded synaptic transmission parameters without cell-type identification. Drug naive mice were used as controls. The distribution and the median value of A/Ns in cocaine SA, NPR mice seemed different from the naive or saline SA, NPR mice, even before grouped data analysis (Fig. 3d). Indeed a high A/N ratio was found in mice self-administering cocaine with NPR, but not in mice self-administering saline or in naive mice (One-way ANOVA: group effect, *F*_(2,28)_ = 33.89, *P* < 0.0001; Tukey: *q*_(19)_ = 7.765, *P* < 0.0001, for A/N in naive *vs* cocaine SA, NPR; Tukey: *q*_(23)_ = 10.79, *P* < 0.0001, for A/N in saline *vs* cocaine SA, NPR) indicating that OFC to DS synapses were strengthened in mice that self-administered cocaine with no punishment risk (Fig. 3e). AMPAR-EPSCs amplitude were higher than NMDAR-EPSCs amplitude in cocaine SA, NPR mice but not in saline SA or naive mice (mixed two-way ANOVA: EPSCs amplitude effect, *F*_(1,28)_ = 10.60, *P* = 0.003; group effect, *F*_(2,28)_ = 6.289, *P* = 0.006; EPSCs amplitude x group effect, *F*_(2,28)_ = 24.85, *P* < 0.0001; Bonferroni post-test: *t*_(14)_ = 8.828, *P* < 0.0001 for AMPA *vs* NMDA in cocaine SA, NPR mice; *t*_(19)_ = 2.951, *P* = 0.014 and *t*_(23)_ = 4.773, *P* < 0.0001 for AMPA-EPSCs in naive vs cocaine SA, NPR and saline *vs* cocaine SA, NPR, respectively) (Fig. 3f). We observed neither rectification nor an alteration in paired pulse ratio (One-way ANOVA: group effect, *F*_(2,28)_ = 1.613, *P* = 0.217 for RI; One-way ANOVA: group effect, *F*_(2,28)_ = 1.397, *P* = 0.264 for PPR) (Fig. 3g). Spontaneous EPSCs (sEPSCs) recorded from the same cells were again not affected (One-way ANOVA: group effect, *F*_(2,28)_ = 2.099, *P* = 0.141 for EPSCs frequency; One-way ANOVA: group effect, *F*_(2,28)_ = 0.922, *P* = 0.409 for sEPSCs amplitude), suggesting a selective potentiation at OFC-DS synapses (Fig. 3h). In summary, we found a mean A/N in mice that self-administered cocaine with no risk of punishment that was higher than those in naive mice or mice self-administering saline but lower than the A/N previously recorded in persevering mice (1.3 vs 1.7), an intermediate value that could reflect a potentiation of lower magnitude or the average of the two populations of SPNs where only one undergoes potentiation.

### Synaptic strengthening in D1R- but not D2R-SPNs after cocaine SA with no punishment risk

**Fig. 4:**
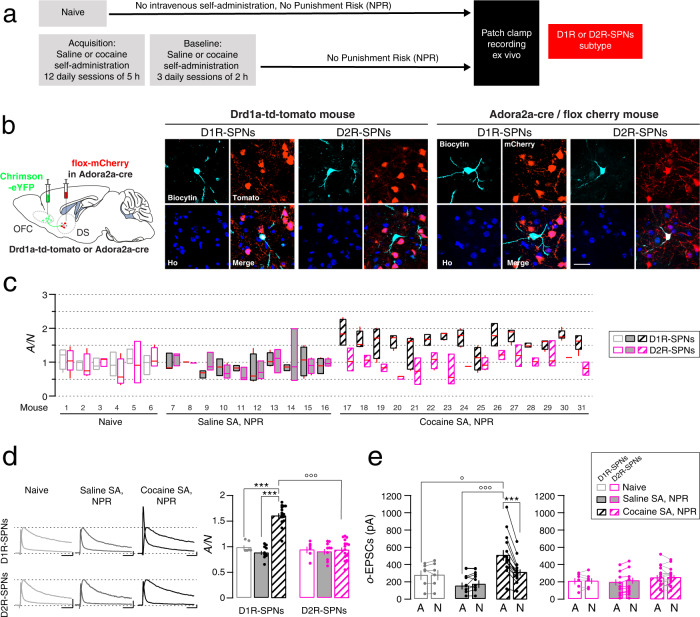
Synaptic potentiation in D1R- but not D2R-SPNs after cocaine SA with no punishment risk. **a** Experiment schedule. **b** Post-hoc identification of direct and indirect pathways SPNs. Slice recordings 24 h after the last baseline session of saline or cocaine SA, NPR or in naive mice infected with Chrimson-eYFP in the OFC. SPNs of the DS from BAC Drd1a- td-tomato transgenic mice and Adora2a-cre mice injected with AAV5Ef1a-DIO-mCherry were filled with biocytin. Examples of neurons considered from the direct pathway (D1R-SPNs) are either positive in Drd1a-td-tomato mouse or negative in Adora2a-cre/flox-cherry mouse. Negative neuron from Drd1a-td-tomato mouse or positive neuron in Adora2a-cre/flox-cherry mouse, conversely considered from the indirect pathway (D2R-SPNs). Scale bar, 50 µm. **c** Box plot of the distribution (median, 25%, 75%, minimal and maximal values) of the ratios of optogentically evoked AMPAR to NMDAR-EPSCs (A/N) recorded at positive potential (+40 mV) from D1R- and D2R-SPNs (total of 120 D1R-SPNs and 95 D2R-SPNs/31 mice, with 1–6 identified cells/mouse) in each in naive and saline or cocaine SA, NPR mice (*N* = 6, 10 and 15 mice respectively). **d** Left, example traces (average of 15 sweeps) of AMPAR- and NMDAR-EPSCs recorded at +40 mV in D1R-or D2R-SPNs from naive and saline or cocaine SA, NPR mice. Scale bars, 50 ms, 50 pA. Right, A/N ratio from D1R-or D2R-SPNs of naive and saline or cocaine SA, NPR (*N* = 6, 10, and 15 mice, total of 120 D1R-SPNs and 95 D2R-SPNs/31 mice, mean of 1-6 identified cells/mouse). Bonferroni post-test ***P < 0.001 for naïve or saline *vs* cocaine SA, NPR in D1R-SPNs; °°°*P* < 0.001 for D1R-SPNs *vs* D2R-SPNs in cocaine SA, NPR. **e** Mean amplitude of AMPAR- and NMDAR-EPSCs evoked with optogenetic stimulation of OFC terminals (o-EPSCs) onto D1R-or D2R-SPNs in slices *per* mouse (*N* = 6, 10, and 15 mice, total of 120 D1R-SPNs and 95 D2R-SPNs/31 mice). Bonferroni post-test ***P < 0.001, for AMPAR vs NMDAR amplitude in D1R-SPNs; Bonferroni post-test °*P* < 0.05, for oAMPAR-EPSCs amplitude in naive vs cocaine SA, NPR; °°°*P* < 0.001, for oAMPAR-EPSCs amplitude in saline *vs* cocaine SA, NPR. Data are expressed as mean ± SEM. Ho Hoechst, NPR no punishment risk.

We next separated D1R- and D2R-SPNs for each mouse to appreciate the cell-by-cell variability and the median of A/N ratio collected (Fig. 4a-b). The distribution and the median value of A/N were similar in D1R and D2R-SPNs for mice that were naive and for mice that underwent saline SA with no punishment risk (Saline SA, NPR mice). By contrast in cocaine SA without punishment risk (Cocaine SA, NPR mice), the distribution of A/N ratios appeared to be higher in D1R compared to D2R-SPNs (Fig. 4c), which was confirmed in the group data (mixed two-way ANOVA: SPNs-type effect, *F*_(1,56)_ = 23.97, *P* < 0.0001; group effect, *F*_(2,56)_ = 33.66, *P* < 0.0001; SPNs-type x group effect, *F*_(2,56)_ = 28.38, *P* < 0.0001; Bonferroni post-test: *t*_(19)_ = 7.43, *P* < 0.0001 and *t*_(23)_ = 10.30, *P* < 0.0001 for cocaine SA, NPR *vs* naive mice and saline SA mice in D1R-SPNs, respectively; *t*_(14)_ = 10.52, *P* < 0.0001 for D1R- vs D2R-SPNs in cocaine NPR mice) indicating that OFC to DS synapses were strengthened after cocaine SA only in D1R-SPNs (Fig. 4d). No such potentiation was observed in mice self-administering saline. The absolute amplitude of AMPAR-EPSCs was larger than NMDAR-EPSCs evoked with optogenetic stimulation in cocaine SA, NPR mice in D1R-SPNs but not in naive or saline SA mice and was also higher in cocaine SA, NPR mice in D1R-SPNs than in naive or saline SA mice (mixed two-way ANOVA: EPSCs effect, *F*_(1,28)_ = 18.36, *P* = 0.0002; group effect, *F*_(2,28)_ = 7.449, *P* = 0.0025; EPSCs-type x group effect, *F*_(2,28)_ = 33.98, *P* < 0.0001; Bonferroni post-test: *t*_(14)_ = 10.75, *P* < 0.0001 for AMPA-EPSCs vs NMDA-EPSCs in cocaine SA, NPR mice; *t*_(19)_ = 2.952, *P* = 0.0138 and *t*_(23)_ = 5.347, *P* < 0.0001 for AMPA-EPSCs naive and saline SA *vs* cocaine SA, NPR mice, respectively). By contrast no differences were found in D2R-SPNs (mixed two way ANOVA: EPSCs effect, *F*_(1,28)_ = 0.85, *P* = 0.847; group effect, *F*_(2,28)_ = 0.84, *P* = 0.842; EPSCs-type x group effect, *F*_(2,28)_ = 0.44, *P* = 0.652). These results suggest that the intermediate increase A/N ratio detected without cell-type identification after acquisition reflects the average between a strong synaptic potentiation onto D1R-SPNs of the DS and no potentiation of the synapses onto D2R-SPNs (Fig. 4e). Taken together, OFC to DS synapses are potentiated after cocaine SA, NPR in D1R-SPNs in every mouse, while synapses onto D2R-SPNs are not modified during cocaine SA without risk of punishment. This result indicates that cocaine evokes synaptic plasticity in D1R-SPNs of the DS, akin to what was repeatedly reported in the NAc.

## Discussion

We monitored the time course of cocaine-evoked synaptic plasticity at OFC projections onto identified SPNs of the DS and observed the following sequence. During acquisition of cocaine SA, OFC afferents onto D1R-SPNs potentiate in all mice. After punishment sessions, OFC afferents onto D2R-SPNs also strengthen, but only in individuals with compulsion-like behavior, while OFC to D1R-SPNs synapses returns to baseline in mice that renounce to cocaine SA with the risk of punishment.

### Methodological and theoretical considerations

Any comparison between individuals persevering and renouncing their drug intake behavior when associated with punishment must control for pre-existing pain sensitivity differences and a potential analgesic effect of the drug. Persevering animals receive more electric shocks, which would be better endured if a higher pain threshold existed. In previous publications [7, 17] we have tested for this possibility and did not find any difference between perseverers and renouncers. We also demonstrated that the A/N ratio at OFC to DS synapses remained unchanged in mice yoked to renouncers or perseverers, showing that foot shocks are not responsible for plasticity observed at these synapses [17].

Another caveat of the model could come from individual differences in the analgesic effects of cocaine, as reported for example in Spontaneously Hypertensive Rats [23]. While we have not directly tested this here, it seems unlikely since the mice used in this study were inbred lines with low genetic variability. An alternate strategy would be to use an aversive punishment stimulus that is not painful, such as an air puff. This will be particularly important when testing opiates with a strong analgesic effect.

Furthermore, it is important to ensure that the mice learned the contingency between foot shock and lever press. For this reason, we delivered the foot shock immediately after the mice pressed the lever. The animals increase their lever presses with an increased fixed ratio and readily distinguish the active from the passive lever. Moreover, even persevering mice reduce their total number of lever presses, at least transiently, supporting the notion that they have learned the contingency. Finally, in an oDASS seek-take chain, the foot shocks were delivered only in unrewarded trials, and the rate of transition to compulsion-like behavior was similar [24], again indicating that mice can readily learn action-outcome contingencies.

We found synaptic strengthening of OFC to DS synapses in both types of striatal SPNs of persevering mice, both with oDASS and cocaine SA, [20]. For oDASS, we causally linked the potentiation of OFC-DS synapses to behavior [17, 25] by applying an in vivo LTD procedure before the punishment sessions, which reduced perseverance. A similar scenario may apply to cocaine SA, where the lower rate of transition to compulsion-like behavior is explained by the opposing role of serotonin on the induction of OFC-DS potentiation [20].

Compulsion-like behavior is observed in several pathological conditions, and addiction models are defined by the perseverance of drug intake despite harmful consequences [26]. In the sophistication of this model, even in the presence of an alternative sucrose reward, some rats were found to endure foot shocks to obtain cocaine intravenous infusions [27]. Our data suggest that compulsion-like behavior is firmly established once the animal experiences punishment.

The synapses onto D2R-SPNs underwent potentiation only in persevering mice after they had to endure punishment to obtain cocaine infusions. It is, therefore, likely that the induction of this form of potentiation was independent of the surge of dopamine evoked by cocaine SA or oDASS. By contrast, we found potentiated synapses in every mouse at the end of the cocaine self-administration procedure, but only in D1R-SPNs. This is reminiscent of the dopamine-dependent synaptic plasticity found in the NAc after cocaine exposure [21, 28].

### Induction mechanism

Distinct rules underlying the induction of synaptic plasticity apply for the two types of SPNs [29, 30]. In SPNs that express D1Rs, crosstalk between D1Rs and glutamate receptors, together with the stimulation of Gα_olf_ (a G protein positively coupled to adenylate cyclase), activates signaling cascades involved in synaptic potentiation such as the intracellular MAPK-ERK pathway [31, 15]. In other words, when afferent activity coincides with increasing DA levels, such as observed in response to addictive drugs, LTP is expressed by the insertion of AMPA receptors in the postsynaptic membrane [10]. In the same conditions, no LTP is induced in D2R-SPNs because D2Rs couple to Gi [29]. Since D2Rs have a high affinity for DA, adenylate cyclase is inhibited at baseline, and brief dips of DA are required to relieve the D2R signaling to induce LTP. The termination of Gi signaling activates PKA, which triggers a cascade of events that eventually strengthen glutamate afferents [32]. Applied to cocaine SA, repeated infusions cause a dopamine transient during acquisition, and LTP is therefore favored at glutamatergic synapses onto D1R-SPNs. During punishment with foot shocks, dopamine levels may dip and thus allow for the induction of synaptic potentiation in D2R-SPNs. There is, however also evidence for increased activity of midbrain DA neurons in response to aversive stimuli [33, 34].

By contrast, the potentiation of glutamatergic inputs onto D2R-MSNs of the NAc has been associated with the resilience of compulsive cocaine seeking [35, 36]. This effect in the NAc, upstream of plasticity in the DS, may reduce the motivation for the drug and thus slow down the progression towards compulsion-like behavior, in agreement with medial prefrontal cortex (mPFC) to NAc exerting behavioral inhibition in punishment-sensitive mice [37].

In addition, the cocaine-evoked potentiation in D1R-SPNs was detected in all mice after acquisition of cocaine SA but returned to baseline after the punishment sessions in renouncing mice. This could be a result of the reduction of cocaine intake in these mice, which may be required to maintain synaptic potentiation through re-exposure to the drug and drug-context. Alternatively, in light of the punishment altered OFC activity (e.g. sustained tonic activity) in renouncing individuals could lead to synaptic depression.

In agreement with the rules of DA modulation of excitatory synapses imposed by the receptors expressed, cocaine exposure causes LTP onto D1R-SPNs while LTP in D2R-SPNs can only occur in a window of decreased DA levels.

### Implications for the search of molecular mechanism underlying vulnerability

Our data may reveal a synaptic mechanism underlying individual vulnerability. “Low levels of dopamine D2 receptors in the striatum” and alteration of cortico-striatal processing have been observed in human addicts [38, 39]. However, D2 receptors are expressed in the striatum by various cell-types. In addition to D2Rs on the soma and dendrites of SPNs and midbrain DA neurons, there are D2Rs on terminals of ascending DA afferents and on the cell body of cholinergic interneurons. Alterations of D2R signaling on any of these cells may cause divergent effects on circuit function. For example, elevated abundance of D2Rs in accumbal cholinergic interneurons was sufficient and necessary for cocaine motivation [40] while loss of D2Rs in midbrain DA neurons may enhance DA neuron activity. The D2R downregulation observed in human drug users therefore would have to be resolved by cell type to establish a prediction on the mechanism underlying enhanced vulnerability.

The reduction of D2R signaling could thus be the cause or the consequence of compulsive drug use, as both scenarios have been observed [41]. Supporting the former, in both rodents and non-human primates, low expression of D2Rs is associated with impulsivity traits that predispose for compulsive cocaine use [42–44]. Others found a decrease in D2R binding only after chronic cocaine exposure in rats [45, 46]. Therefore, in individuals who express fewer dopamine D2R in striatal neurons, LTP could be induced more readily in D2R-SPNs, and compulsion-like behavior arises quicker [47]. Beyond the number of D2Rs, it is also possible that dynamic regulation of Gi signaling *via* RGS proteins [48] can contribute to the transition to compulsive cocaine use by promoting LTP in these cells.

Taken together, OFC to DS synapses are potentiated after cocaine SA in D1R-SPNs in every mouse, while synapses onto D2R-SPNs are strengthened only in persevering mice when transitioning to compulsion-like cocaine use during the punishment sessions is achieved. Our data provide a comprehensive picture of the steps and cell type determinants of the neural adaptations associated with compulsion-like behavior. These elements will significantly facilitate the search for the molecular underpinnings of individual addiction vulnerability.

## Supplementary information

Supplemental Material
