# Supplementary material for: Cell-type specific synaptic plasticity in dorsal striatum is associated with punishment-resistance compulsive-like cocaine self-administration in mice
Source: Neuropsychopharmacology. 2022 Sep 7;48(3):448–58. doi: 10.1038/s41386-022-01429-8 (PMC9852591; doi:10.1038/s41386-022-01429-8)
Supplement: Supplementary file 1 — Supplemental Material [file 41386_2022_1429_MOESM1_ESM.pdf]

SUPPLEMENTAL MATERIAL

Cell-type specific synaptic plasticity in dorsal striatum is associated with punishment-resistance compulsive-like cocaine self-administration in mice

Vincent Pascoli[1,3], Agnès Hiver[1,3], Yue Li[1], Masaya Harada[1], Vahid Esmaeili[1] and Christian Lüscher[1,2]

[1] Department of Basic Neurosciences, Medical Faculty, University of Geneva, 1211 Geneva, Switzerland

[2] Clinic of Neurology, Department of Clinical Neurosciences, Geneva University Hospital, 1211 Geneva, Switzerland

[3] Co-first author

*Correspondence: Christian.Lüscher@unige.ch

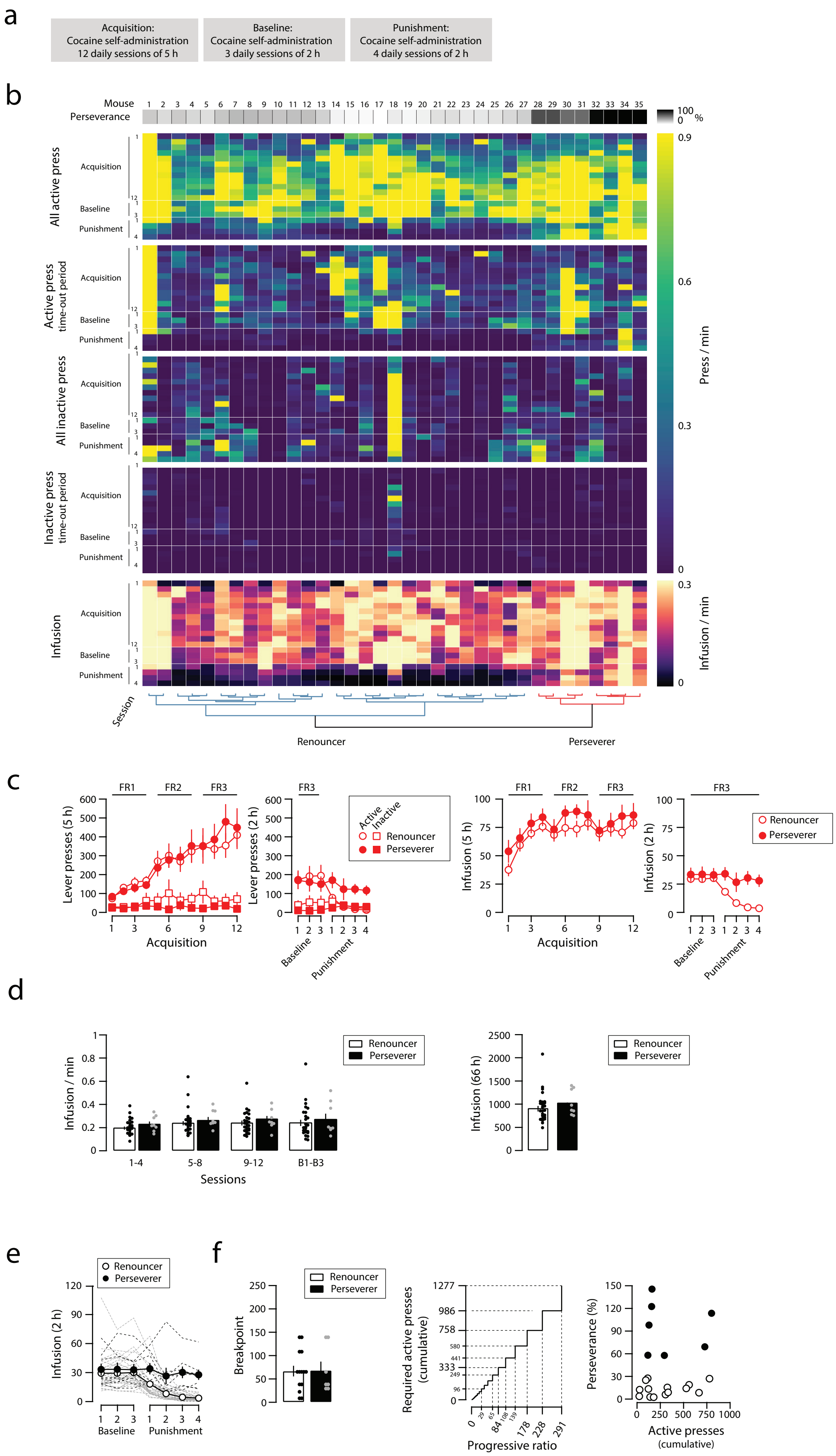

Pascoli et al., Supplemental Figure 1

Supplemental Figure 1: Retrospective analysis of cocaine self-administration acquisition in renouncing and persevering mice.

(a) Experiment schedule. **(b)** Heat maps showing the cocaine self-administration behavioral parameter of each mouse during acquisition, baseline and punished sessions. Clustering was performed with all behavioral parameters of the last two punished sessions and correlation with perseverance. **(c)** Active and inactive lever presses (left) and infusion (right) during acquisition, baseline and punished sessions of cocaine self-administration for renouncing and persevering mice ($N = 27$ and 8 mice, respectively). **(d)** Left, Mean rate of infusions during the different phases of cocaine self-administration (4 sessions of 5 h with FR1 (1-4), 4 sessions of 5 h with FR2 (5-8), 4 sessions of 5 h with FR3 (9-12) and the 3 baseline sessions of 2 h with FR3 (B1-B3)). Right, Total number of infusions obtained during the 15 sessions of cocaine self-administration before the introduction of punishment in renouncers and perseverers ($N = 27$ and 8 mice, respectively). The time spent during these 15 sessions represent 66 hours. **(e)** Mean number of cocaine infusions during to baseline and punished sessions in renouncers and perseverers ($N = 27$ and 8 mice respectively). Dashed lines represented individual mouse. **(f)** Left, Breakpoint for renouncers and perseverers ($N = 14$ and 7 mice, respectively). Middle, schedule of the progressive ratio showing the cumulative number of active lever presses required to reach each reinforced ratio. Right, Perseverance as a function of cumulative number of active presses made during the progressive ratio session. Each dots represents a renouncer or a perseverer ($N = 14$ and 7 mice respectively). Data are expressed as mean ± SEM.

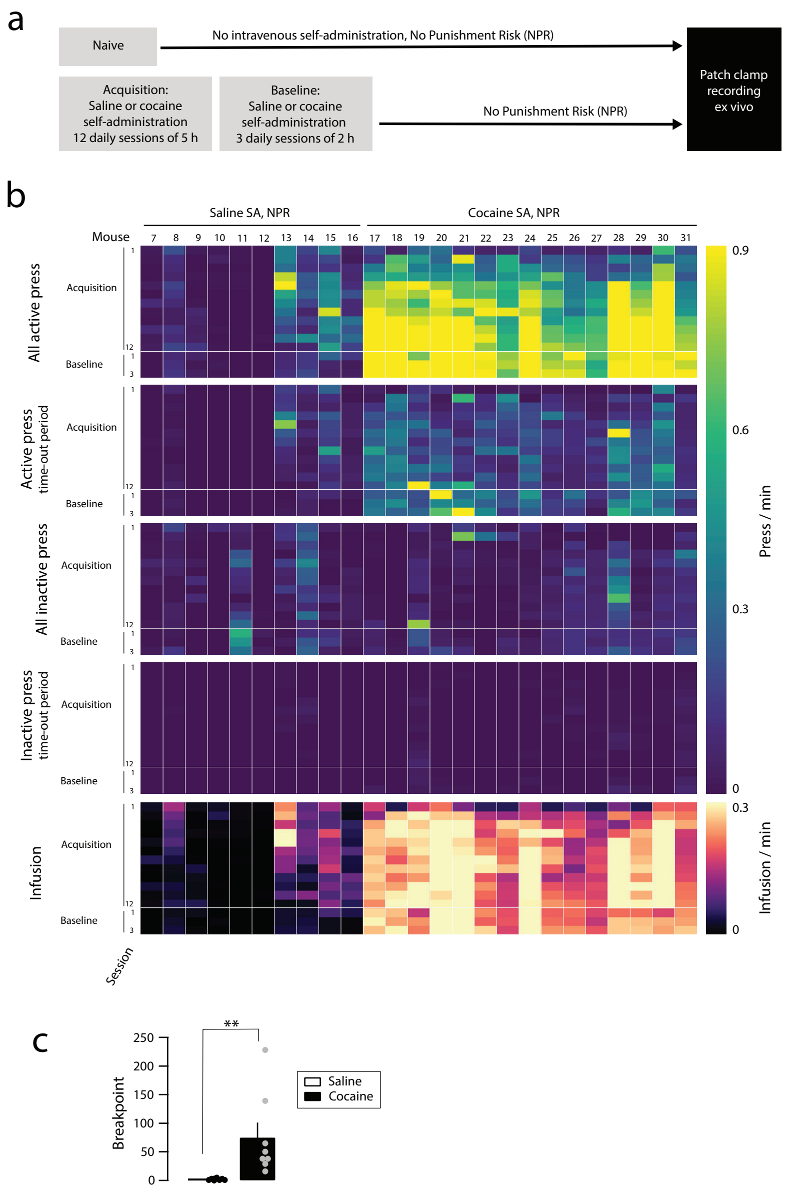

Pascoli et al., Supplemental Figure 2

Supplemental Figure 2: Cocaine *vs* saline intravenous self-administration

(a) Experiment schedule. **(b)** Heat maps showing the saline and cocaine self-administration behavioral parameter of each mouse during acquisition and baseline sessions. **(c)** Breakpoint obtained during the progressive ratio experiment mice self-administering saline or cocaine (N = 10 and 8 mice, respectively). Student's t-test: $^{**}P<0.01$. Data are expressed as mean ± SEM.
